# Higher Gene Expression Of Dynein Heavy Chains In The Dorsolateral Prefrontal Cortex Predict Lower Neuropathology Burden And Better Cognitive Outcomes In Individuals With Alzheimer’s Disease

**DOI:** 10.1002/alz.091614

**Published:** 2025-01-03

**Authors:** Esteban M Lucero, Tonya Brunetti, Heidi J Chial, Huntington Potter, Chris Gignoux

**Affiliations:** ^1^ University of Colorado, Anschutz Medical Campus, Aurora, CO USA; ^2^ University of Colorado Alzheimer’s and Cognition Center, Aurora, CO USA; ^3^ Linda Crnic Institute for Down Syndrome, Aurora, CO USA; ^4^ University of Colorado Anschutz, Aurora, CO USA; ^5^ Colorado Center for Personalized Medicine, Aurora, CO USA

## Abstract

**Background:**

Motor proteins play a key role in neuronal functions and morphology that are important for learning and memory. We have previously reported that increased expression *KIF11/*Kinesin‐5 overrides Aß‐mediated effects on dendritic spine density and long‐term potentiation in a mouse model of Alzheimer’s disease (AD), effectively maintaining cognitive function in the face of Aß pathology. Here, we evaluated the association of key AD phenotypes with mRNA expression levels of a select set of Dynein motor proteins

**Method:**

We utilized measurements of gene expression, AD neuropathology burden, and cognition provided by the ROS/MAP study to determine whether an association exists between AD phenotypes and expression of genes for cytoplasmic and axonemal dynein heavy chains. Neuropathology burden was determined through immunohistochemistry. Z‐scores from the raw scores of 19 cognitive tests were used to determine global cognition. Neuropathology burden and global cognition measurements were provided by Rush Alzheimer’s Disease Center. Measurements of gene expression in the dorsolateral prefrontal cortex (DLPFC) (n = 634) were determined through an established RNAseq analysis pipeline (Logsdon et al., 2019, syn8456638;syn8456629) and made available to us through the Accelerating Medicines Partnership in Alzheimer’s Disease Target Discovery and Preclinical Validation knowledge portal (syn3219045). Associations of gene expression with neuropathology and cognition were determined through multiple linear regression and mixed effects models covarying for age, sex, education, and post‐mortem interval

**Result:**

In participants with AD (CERAD score > 2), higher gene expression levels of *DYNC1H1* and *DNAH1* in the DLPFC predicted better cognitive performance longitudinally (p = 0.03 and p = 0.00197, respectively) and at the last visit prior to death (p = 8.701e‐05 and p = 3.571e‐05, respectively). Higher expression of *DYNC1H1* and *DNAH1* were also associated with lower amyloid pathology (p = 0.0001257 and p = 2.356e‐07, respectively) and tau tangles (p = 2.356e‐07 and p = 2.659e‐09, respectively)

**Conclusion:**

Our finding that AD participants with higher expression levels of *DYC1H1* and *DNAH1* show reduced cognitive decline and decreased AD pathology suggest a potential beneficial effect of these motor proteins on AD progression. Based on the functions of these motor proteins, these findings warrant further studies to identify potential mechanisms underlying this association. Potential mechanisms include facilitation of higher rates of phagocytosis or neuronal lysosomal degradation of Aß

